# Supplementary material for: Rhododendron luteum Sweet Flower Supercritical CO2 Extracts: Terpenes Composition, Pro-Inflammatory Enzymes Inhibition and Antioxidant Activity
Source: Int J Mol Sci. 2024 Sep 15;25(18):9952. doi: 10.3390/ijms25189952 (PMC11432528; doi:10.3390/ijms25189952)
Supplement: Supplementary file 1 [file ijms-25-09952-s001.zip › ijms-3176104-supplementary.pdf]

Supplementary Material

Rhododendron luteum Sweet Flower Supercritical CO_2 Extracts: Terpenes Composition, Pro-Inflammatory Enzymes Inhibition and Antioxidant Activity

Lena Łyko [1], Marta Olech [1], Urszula Gawlik [2], Agnieszka Krajewska [3], Danuta Kalemba [3], Katarzyna Tyśkiewicz [4], Narcyz Piórecki [5,6], Andriy Prokopiv [7] and Renata Nowak [1,*]

Table S1. LC gradient details used during LC-MS analysis of triterpenes.

Time (min)	Solution A (%)	Solution B (%)
0	68	32
5	68	32
6	62	38
12	62	38
18	56	44
21	56	44
26	43	57
31	43	57
32	38	62
41	38	62
42	30	70
47	30	70
52	5	95
63	5	95
66	0	100
72	0	100
75	68	32
85	68	32

Table S2. Summary of optimized parameters for the quantitative analysis of triterpenes.

Compound	Retention time (min)	Polarity	Precursor ion (m/z)	Fragment ions (m/z)	Collision energy (V)
Arjungenin	7.78	neg	503.3	503.3<br>409	-17<br>-46.9
Euscaphic acid	16.84	neg	487.3	487.3<br>455.4	-27.7<br>-27.5
Arjunic acid	23.41	pos	471.5	105.2<br>91.1	85.9<br>104.5
Maslinic acid	29.95	neg	471.3	471.3<br>423.1	-22.3<br>-63.4
Corosolic acid	30.76	neg	471.4	471.4<br>423.3	-24.5<br>-36.9
Betulinic acid	42.7	pos	439	95<br>81	54<br>57.7
Ursolic acid	43.82	neg	455.4	455.4<br>407.3	-22.2<br>-51
Oleanolic acid	43.65	pos	439	91.2<br>119.1	108.6<br>63
Erythrodiol	47.62	pos	425.4	95.2<br>81.2	50.6<br>67.1
Uvaol	47.90	pos	425.3	95.2<br>81.2	53<br>69
Lupeol	62.86	pos	409.6	95.21<br>81.2	50.5<br>61.5
3β-Taraxerol	64.72	pos	409.4	95.3<br>81.2	53.2<br>56.7
α-Amyrin	65.96	pos	409.6	367.4<br>409.6	27.8<br>25.1
β-Sitosterol	68.85	pos	397.6	91.1<br>105.1	95.1<br>66.8

Table S3. Analytical parameters of LC-MS/MS quantitative method for determination of triterpenes.

Compound	LOD [μg/mL]	LOQ [μg/mL]	Linearity range [ng/mL]	R^2
Euscaphic acid	0.40	0.8	1000-60000	0.9989
Maslinic acid	0.60	1.00	1000-5000	0.9981
Corosolic acid	0.3125	0.625	625-20000	0.9992
Betulinic acid	0.625	1.25	1250-20000	0.9993
Ursolic acid	0.15	0.31	313-20000	0.9996
Oleanolic acid	1.05	1.80	1800 - 50000	0.9999
Erythrodiol	0.2	0.313	313-20000	0.9993
Uvaol	0.625	1.25	1560 - 50000	0.9992
Lupeol	0.20	0.32	313-20000	0.9996
3*β*-Taraxerol	0.625	1.25	313-20000	0.9984
α-Amyrin	0.5	1.13	1560 - 50000	0.9995
β-Sitosterol	0.45	0.80	800-20000	0.9993
